# Higher perioperative dexamethasone exposure is associated with shorter survival in glioblastoma

**DOI:** 10.1093/noajnl/vdag099

**Published:** 2026-04-13

**Authors:** Severin Rüssli, Claire Descombes, Alexis P R Terrapon, Manuel Hinsberger, Theoni Maragkou, Irena Zubak, Levin Häni, Philippe Schucht, Andreas Raabe, Johannes Goldberg

**Affiliations:** Department of Neurosurgery, Inselspital, Bern University Hospital, University of Bern, Bern, Switzerland; Department of Neurosurgery, LUKS, Cantonal Hospital of Lucerne, Lucerne, Switzerland; Department of Neurosurgery, Inselspital, Bern University Hospital, University of Bern, Bern, Switzerland; Department of Neurosurgery, Inselspital, Bern University Hospital, University of Bern, Bern, Switzerland; Department of Neurosurgery, Inselspital, Bern University Hospital, University of Bern, Bern, Switzerland; Department of Neurology, University Hospital Frankfurt, Goethe University, Frankfurt am Main, Germany; Institute of Tissue Medicine and Pathology, University of Bern, Bern, Switzerland; Department of Neurosurgery, Inselspital, Bern University Hospital, University of Bern, Bern, Switzerland; Department of Neurosurgery, Inselspital, Bern University Hospital, University of Bern, Bern, Switzerland; Department of Neurosurgery, Inselspital, Bern University Hospital, University of Bern, Bern, Switzerland; Department of Neurosurgery, Inselspital, Bern University Hospital, University of Bern, Bern, Switzerland; Department of Neurosurgery, Inselspital, Bern University Hospital, University of Bern, Bern, Switzerland

**Keywords:** dexamethasone, glioblastoma, glucocorticoids, survival

## Abstract

**Background:**

Dexamethasone (DEX) is routinely administered perioperatively to manage tumor-associated vasogenic edema in glioblastoma (GBM), yet increasing evidence suggests that corticosteroid exposure may adversely affect survival. The magnitude of this association during the initial neurosurgical phase of care remains unclear.

**Methods:**

We performed a retrospective cohort study of patients with histologically confirmed IDH-wildtype GBM treated at a single tertiary center between 2009 and 2020. All perioperative DEX doses from admission to discharge were extracted from daily medical records. Patients were stratified based on the cumulative dose into low-dose exposure (<34 mg) and high-dose exposure (≥34 mg) groups using maximally selected rank statistics. Overall survival was analyzed using Kaplan-Meier estimates with log-rank test and multivariable Cox proportional hazards models. Adjustment variables were selected using a prespecified, causally informed framework to address confounding.

**Results:**

A total of 420 patients were included. The majority (*n* = 341; 81.2%) received ≥34 mg DEX perioperatively. Median OS was 13.6 months in the high-dose group and 15.0 months in the low-dose group, with significantly shorter survival observed in the high-dose cohort (log-rank *P* = .0103). In the adjusted Cox model, cumulative DEX ≥ 34 mg remained independently associated with increased mortality (HR: 1.40, 95% CI: 1.07-1.83; *P* = .013).

**Conclusions:**

Higher perioperative DEX doses were independently associated with shorter overall survival in GBM patients, emphasizing the need for judicious perioperative use with prompt tapering. Prospective studies are warranted to guide evidence-based DEX management in GBM care.

Key Points

- High perioperative dexamethasone exposure (≥34 mg) is independently associated with shorter overall survival in glioblastoma.
- Minimizing perioperative dexamethasone doses may improve survival outcomes.

Importance of the Study

Recent studies have suggested a negative association between DEX use and overall survival in GBM. This study is the first to systematically evaluate cumulative perioperative DEX exposure during the neurosurgical hospitalization and its impact on survival. We identified a cumulative perioperative dose of ≥34 mg as a relevant threshold independently associated with shorter overall survival. Because DEX is a long-established component of perioperative glioblastoma management, and is frequently administered at high doses during this critical phase, these findings offer a clinically meaningful benchmark for guiding steroid use. Our results underscore the importance of minimizing cumulative DEX exposure whenever feasible and support the need for prospective studies to establish evidence-based dosing strategies that balance symptom control with potential risks.

The administration of glucocorticoids, particularly dexamethasone (DEX), for the treatment of tumor-induced vasogenic cerebral edema in glioblastoma (GBM) is a well-established clinical practice since the 1960s.^1^ In patients in whom such edema causes mass effect, elevated intracranial pressure, or focal neurological deficits, the administration of DEX can lead to significant clinical improvement by reducing edema and lowering intracranial pressure.^2^^,^^3^ Beyond its perioperative use, DEX also plays a relevant role in the adjuvant management of GBM. The antiedematous and antiemetic effects of DEX have been demonstrated in several studies, establishing its use for symptom control and the mitigation of side effects associated with radiochemotherapy.^4^^,^^5^ Because of its long-established role in GBM care, DEX is often initiated by primary care providers, referring physicians, or neurosurgeons when GBM is suspected on brain imaging, even in patients with only mild edema or minor neurological deficits, where its use may not be necessary. Once started, treatment is often continued for prolonged periods, even when the ­clinical need diminishes.

The prognosis for patients diagnosed with GBM remains poor.^6^ Several studies have suggested that DEX and other glucocorticoids may negatively affect survival.^7–11^ Possible explanations for this include the suppression of antitumor immune responses and modulation of the tumor microenvironment,^8^^,^^12–15^ the interference with the efficacy of therapeutic approaches,^16–18^ as well as general complications resulting from DEX treatment.^19–22^ However, the true underlying effect of DEX on survival in GBM remains incompletely understood. Establishing causal inference is challenging as numerous clinical and treatment-related variables strongly influence survival and act as major confounders. In particular, patients with poorer clinical status, larger tumors, or those not undergoing resection are more likely to receive DEX. Given these uncertainties and the need for additional data, particularly in the perioperative neurosurgical setting, we aimed to refine the current evidence by examining the association between perioperative DEX exposure and overall survival of GBM patients in a well-characterized institutional cohort.

## Methods

### Study Design

We conducted a retrospective cohort study using our institutional glioma database, comprising patients with the first diagnosis of GBM, IDH-wildtype, CNS WHO grade 4 treated at Inselspital, Bern University Hospital, Switzerland, over a 12-year period.

### Patient Population

The study included all consecutive patients with a primary diagnosis of GBM treated at our institution between January 2009 and December 2020. Diagnoses were established according to the WHO classification system valid at the time of diagnosis. Cases with indeterminate IDH mutation status were reviewed by a senior neuropathologist. In accordance with the 2021 WHO classification of tumors of the central nervous system,^23^ cases with IDH1/2 mutations or missing IDH status were excluded.

A complete-case analysis was performed for the primary estimand (total effect model), restricting the cohort to patients with comprehensive medical records containing sufficient information on perioperative medication use and complete data for all covariables in the pre-exposure adjustment set. In addition, patients receiving established long-term glucocorticoid therapy for conditions other than GBM were excluded. For the complementary direct-effect model, multiple imputation was applied only to additionally required covariates with missingness (post-exposure variables), while exposure and outcome definitions remained unchanged.

### Data Collection and Outcome Measure

Daily medical records of all patients from referral until discharge from the neurosurgical department were reviewed. All administered perioperative DEX-doses, including intraoperative administration, were extracted. In the rare instances where glucocorticoids other than DEX were given, each dose was converted to its DEX-equivalent based on standard potency ratios.^24^ The exposure of interest was the total cumulative DEX-equivalent dose administered during hospitalization, defined as the sum of all doses from the day of admission until discharge.

Pre- and postoperative tumor volumes were measured using the hospital’s internal picture archiving and communication system IDS7 (Version 25.2, Sectra AB, Linköping, Sweden). Postoperative residual tumor volume was assessed using 1mm subtraction MRI sequences (contrast-enhanced T1 minus native T1). When subtraction images were unavailable, 1 mm contrast-enhanced T1-weighted sequences in combination with native T1-weighted images were used to distinguish residual enhancing tumor from postoperative hemorrhage within the resection cavity. Based on these volumetric measurements, extent of resection (EOR) was categorized as follows: biopsy (<50% tumor removal), debulking (≥50% to <95%), near total resection (≥95% to <99%), or gross total resection (≥99%). For patients who underwent an initial biopsy followed by subsequent resection, EOR was based on the procedure that achieved the maximal tumor removal (resection).

Further, we recorded patients’ demographic and clinical characteristics, including age at surgery, sex, tumor focality, length of stay, type of adjuvant therapy, and perioperative complications (any, neurological, and systemic). Patients’ clinical status was assessed using the Glasgow Coma Scale (GCS) at admission and the Karnofsky Performance Scale (KPS) at both admission and discharge.

The primary estimand was the total effect of cumulative perioperative DEX exposure on overall survival (OS).

### Statistical Analysis

R version 4.5.1 “Great Square Root” (R Foundation for Statistical Computing, Vienna, Austria) and RStudio Version 2025.05.1 (Posit, Boston, MA, United States) were used for computing descriptive and causal inference analyses.

To establish an optimal cut-off value for cumulative perioperatively administered DEX doses, we employed maximally selected rank statistics using the maxstat R package (version 0.7-26) with *P-*value correction after Hothorn & Lausen.^25^ The cohort was subsequently divided into 2 groups: a high-dose exposure group and a low-dose exposure group.

Descriptive statistical analyses were conducted to summarize demographic and baseline characteristics of both groups. Continuous variables were assessed for normality using Q-Q plots. Continuous variables are presented as mean ± standard deviation (SD) or median (IQR), and categorical variables as counts (percentages). Group comparisons used Welch’s two-sample t-test, Wilcoxon rank-sum test, Pearson’s chi-square test, or Fisher’s exact test, as appropriate. *P* values were adjusted for multiple testing using the Holm-Bonferroni correction method. For survival analysis, OS was calculated from the date of first surgery to the date of death. Patients still alive at the time of data collection were censored at their last known follow-up. To estimate OS stratified by DEX exposure (low-dose vs high-dose groups), Kaplan-Meier survival curves were plotted, and group differences were assessed for significance using a log-rank test.

**Figure 1.**
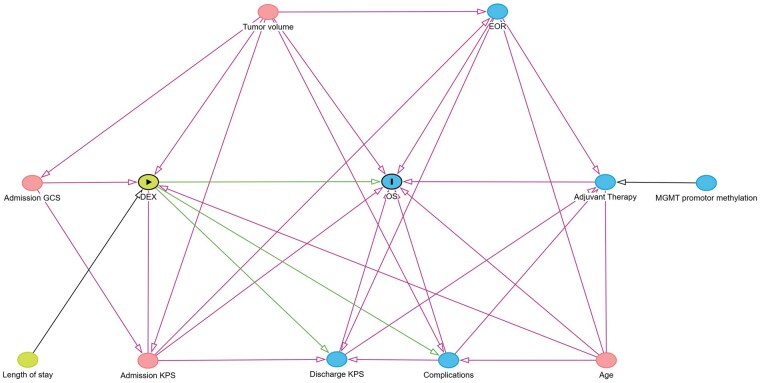
Directed acyclic graph. Nodes are color-coded according to their role in the model: the exposure is shown in green with a triangle symbol and black border; the outcome in blue with a bar symbol and black border; ancestors of the exposure in green; ancestors of the outcome in blue; and variables that are ancestors of both exposure and outcome in red. Green arrows denote causal pathways, whereas red arrows represent biasing (non-causal) paths. Abbreviations: DEX, dexamethasone; OS, overall survival; EOR, extent of resection; KPS, Karnofsky Performance Score; GCS, Glasgow Coma Scale.

Causal survival analyses were prespecified using a directed acyclic graph (DAG) (Figure 1) and implemented with Cox proportional hazards models. Two estimands were evaluated: (1) the total effect of DEX on OS (primary estimand), adjusted for the pre-exposure set (admission KPS, age at surgery, preoperative tumor volume), and (2) a complementary direct effect model additionally adjusting for selected downstream variables (discharge KPS, EOR, any complications). The total-effect model was estimated as complete-case analysis. For the direct-effect model, multiple imputation by chained equations was applied to additionally required covariates with missingness (particularly discharge KPS), while exposure and outcome definitions were unchanged. Proportional hazards assumptions were evaluated using Schoenfeld residuals. Age demonstrated nonproportionality and was modeled with a time-varying interaction term (age × log(time)) in both models. In the direct-effect model, additional covariates showed evidence of non-proportional hazards; therefore, sensitivity analyses were conducted using time-varying terms and stratification where appropriate. The exposure variable (DEX) satisfied the proportional hazards assumption in all analyses. Survival analyses were performed using the survival (Version 3.8-3) and survminer (Version 0.5.1) packages.^26^ Missing covariate data in the direct-effect model were handled using multiple imputation by chained equations with the mice package (*m* = 20 imputed datasets).^27^ The DAG was constructed using dagitty.net and the dagitty R package.^28^ The full DAG specification code is provided in Supplementary Material S1 to ensure transparency and reproducibility. Statistical significance was set at a two-sided alpha level of 0.05 for all analyses.

## Results

### Study Population

**Figure 2.**
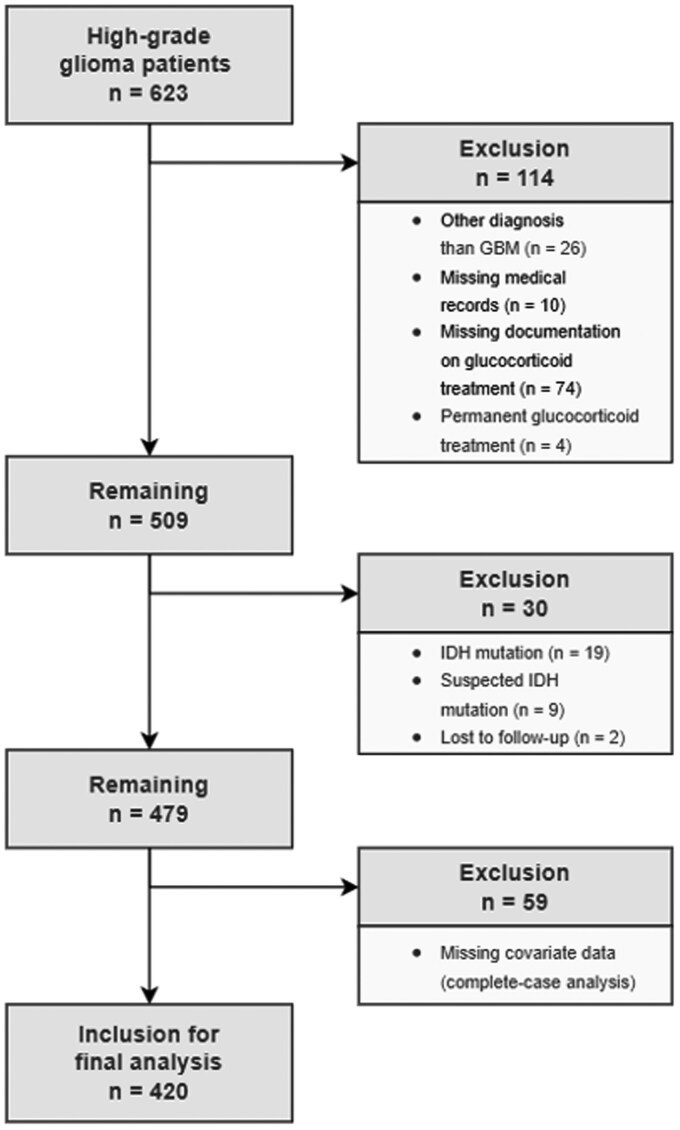
Study flow-chart.

Between January 2009 and December 2020, a total of 623 patients with a primary diagnosis of high-grade glioma were recorded in our institutional glioma registry. For the present analysis, 114 patients were initially excluded: 26 due to diagnoses other than GBM, 10 owing to missing medical records, 74 because of missing documentation of DEX treatment, and 4 due to established long-term DEX therapy for conditions unrelated to GBM. Among the remaining 509 patients, a further 30 were excluded: 19 with confirmed IDH mutations and 9 with initially uncertain IDH status who, after review by a senior neuropathologist, were reclassified as suspected IDH-mutant but could not be reanalyzed due to missing tissue. Two additional cases were excluded due to loss to follow-up. Of the resulting 479 cases, 59 were excluded due to missing data in predefined covariates required for the total-effect model. The final study cohort therefore comprised 420 patients (Figure 2).

### Baseline Characteristics

Patients had a mean age of 64.9 years (SD: ± 11.1) and were predominantly male (n = 282; 67.1%). Maximally selected rank statistics identified an optimal cut-off value for the cumulative perioperatively administered DEX dose at 34mg. Based on the identified cut-off value, 341 patients (81.2%) were classified into the high-dose group (DEX ≥34 mg), and 79 patients (18.8%) into the low-dose group (DEX <34 mg). All continuous variables demonstrated normal distributions on visual assessments. Patients receiving high-dose DEX had lower admission KPS and greater preoperative tumor volumes than those in the low-dose group. Other baseline characteristics were largely similar between groups. Perioperatively, the high-dose group demonstrated longer length of stay and a higher rate of any complications (Table 1). A detailed breakdown of all recorded complications is provided in Supplementary Material S2.

**Table 1.** Baseline characteristics

	Full cohort *N *= 420^1^	Low-dose (<34 mg) *N* = 79^1^	High-dose (≥34 mg) *N* = 341^1^	Adjusted *P* value^2^
Age at surgery (years)	64.9 ± 11.1	65.9 ± 12.3	64.7 ± 10.8	>.99
Sex				>.99
Male	282 (67.1%)	55 (69.6%)	227 (66.6%)	
Female	138 (32.9%)	24 (30.4%)	114 (33.4%)	
GCS at admission (points)				.41
15	347 (82.6%)	72 (91.1%)	275 (80.6%)	
13-14	69 (16.4%)	6 (7.6%)	63 (18.5%)	
<13	4 (1.0%)	1 (1.3%)	3 (0.9%)	
KPS at admission (%)				**.006**
90-100 (≙ ECOG 0)	168 (40.0%)	47 (59.5%)	121 (35.5%)	
70-80 (≙ ECOG 1)	202 (48.1%)	26 (32.9%)	176 (51.6%)	
<70 (≙ ECOG ≥ 2)	50 (11.9%)	6 (7.6%)	44 (12.9%)	
KPS at discharge (%)				.41
90-100 (≙ ECOG 0)	144 (42.7%)	38 (55.9%)	106 (39.4%)	
70-80 (≙ ECOG 1)	146 (43.3%)	22 (32.4%)	124 (46.1%)	
<70 (≙ ECOG ≥ 2)	47 (13.9%)	8 (11.8%)	39 (14.5%)	
Missing	83 (20%)	11 (14%)	72 (21%)	
Tumor focality				.81
Unifocal	369 (87.9%)	65 (82.3%)	304 (89.1%)	
Multifocal	51 (12.1%)	14 (17.7%)	37 (10.9%)	
Tumor volume (cm³)	37.9 ± 30.1	27.2 ± 30.1	40.3 ± 29.6	**.008**
Extent of resection				.67
Biopsy	101 (24.0%)	27 (34.2%)	74 (21.7%)	
Debulking	84 (20.0%)	12 (15.2%)	72 (21.1%)	
Near total resection	65 (15.5%)	9 (11.4%)	56 (16.4%)	
Gross total resection	170 (40.5%)	31 (39.2%)	139 (40.8%)	
MGMT promoter methylation	183 (43.6%)	37 (46.8%)	146 (42.8%)	>.99
Adjuvant therapy				>.99
No adjuvant therapy	38 (9.0%)	7 (8.9%)	31 (9.1%)	
Chemotherapy alone	3 (0.7%)	0 (0.0%)	3 (0.9%)	
Radiotherapy alone	36 (8.6%)	5 (6.3%)	31 (9.1%)	
Combined	343 (81.7%)	67 (84.8%)	276 (80.9%)	
Length of stay (days)	9 (7-13)	7 (5-11)	10 (7-14)	**<.001**
Any complications	109 (26.6%)	9 (12.0%)	100 (29.9%)	**.028**
Missing	10 (2.4%)	4 (5.1%)	6 (1.8%)	
Neurological complications	73 (17.8%)	6 (8.0%)	67 (20.0%)	.22
Missing	10 (2.4%)	4 (5.1%)	6 (1.8%)	
Systemic complications	56 (13.7%)	6 (8.0%)	50 (15.0%)	.81
Missing	12 (2.9%)	4 (5.1%)	8 (2.3%)	
Total DEX dose (mg)	75.6 ± 44.8	19.5 ± 10.4	88.7 ± 39.3	**<.001**

1 Mean ± SD; *n* (%); median (Q1-Q3).

2 Welch Two-Sample *t*-test; Pearson’s Chi-squared test; Fisher’s exact test; Wilcoxon rank sum test; Holm-Bonferroni adjusted *P*-values are displayed to account for multiple testing. Bold values indicate statistically significant Holm-Bonferroni adjusted *P*-values (*P* < 0.05).

Observed data only (no imputation). Continuous variables are presented as mean ± SD or median (IQR); categorical variables as *n* (%).

Tests: Welch *t*-test (age, tumor volume, and total DEX dose); Pearson chi-square (sex, KPS admission, KPS discharge, tumor focality, EOR, MGMT, and complications); Fisher’s exact test (GCS admission, adjuvant therapy); Wilcoxon rank-sum (length of stay).

### Survival Analysis

**Figure 3.**
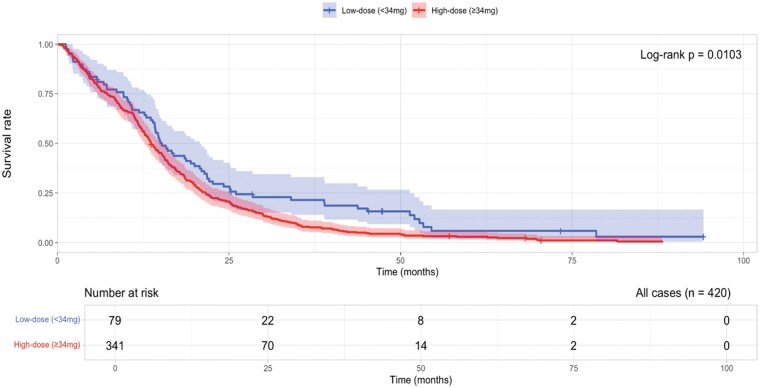
Kaplan-Meier curve by DEX cut-off (34 mg).

The median OS for the entire cohort was 14.1 months (95% CI: 13.2-15.2 months). Patients in the low-dose group (<34 mg DEX) had a median OS of 15.0 months (95% CI: 13.9-20.7 months), whereas those in the high-dose group (≥34 mg DEX) had a median OS of 13.6 months (95% CI: 12.7-15.2 months). The Kaplan-Meier curves showed shorter OS in the high-dose group compared with the low-dose group, which was confirmed by the Log-Rank test (*P* = .0103) (Figure 3).

In the primary total-effect model, high perioperative DEX exposure (≥34 mg) was significantly associated with a higher hazard of death (HR: 1.40, 95% CI: 1.07-1.83; *P* = .013), after adjustment for the DAG-derived preexposure confounders admission KPS, age at surgery, and tumor volume. Age demonstrated a significant time-dependent association with mortality. While increasing age was associated with higher hazard at baseline (HR: 1.10 per year, 95% CI: 1.07-1.14; *P* < .001), the age × log(time) interaction term (HR: 0.97, 95% CI: 0.96-0.98; *P* < .001) indicated attenuation of this effect over follow-up rather than a constant proportional effect. This time-varying pattern is visualized in an HR plot in Supplementary Material S3. Admission KPS was independently associated with improved survival (HR: 0.99 per point increase, 95% CI: 0.98-1.00; *P* = .008), whereas preoperative tumor volume was not significantly associated with mortality (HR: 1.00; *P* = .886).

In the complementary direct-effect model, which additionally adjusted for discharge KPS, EOR, and any perioperative complication, high perioperative DEX exposure (≥34 mg) remained significantly associated with increased mortality (HR: 1.60, 95% CI: 1.21-2.10; *P* < .001). Admission KPS was no longer significantly associated with survival (HR: 1.00, 95% CI: 0.99-1.01; *P* = .995), and tumor volume remained non-significant (HR: 1.00, 95% CI: 1.00-1.01; *P* = .400). The time-dependent effect of age was unchanged compared with the total effect model. Discharge KPS was independently associated with improved survival (HR: 0.99 per point increase, 95% CI: 0.98-1.00; *P* = .018). EOR was strongly protective versus biopsy (debulking: HR: 0.59, 95% CI: 0.43-0.81, *P* = .001; near-total resection: HR: 0.45, 95% CI: 0.32-0.65, *P* < .001; gross-total resection: HR: 0.40, 95% CI: 0.30-0.53, *P* < .001).

The presence of any complication showed a directionally increased hazard without reaching statistical significance (HR: 1.23, 95% CI: 0.97-1.56; *P* = .093) (Table 2). Proportional hazards were reassessed in the direct-effect model. While DEX satisfied the assumption, selected adjustment covariates showed nonproportionality. In sensitivity analyses using time-varying terms and stratification, the DEX-mortality association remained stable (HR: 1.50), supporting robustness of the findings.

**Table 2.** Cox proportional hazards models for overall survival evaluating perioperative high-dose DEX exposure (≥34 mg), presented as a total-effect model (primary estimand) and a complementary direct-effect model

	Total effect model *N* = 420, no imputation		Direct effect model *N* = 420, MI (m = 20)	
	HR (95% CI)	*P*	HR (95% CI)	*P*
DEX high-dose (≥34 mg) vs. low-dose (<34 mg)	**1.40 (1.07-1.83)**	**.013**	**1.60 (1.21-2.10)**	**<.001**
Admission KPS (per point)	0.99 (0.98-1.00)	.008	1.00 (0.99**-**1.01)	.995
Tumor volume (per mL)	1.00 (1.00-1.00)	.886	1.00 (1.00**-**1.01)	.400
Age at surgery (per year)	1.10 (1.07-1.14)	<.001	1.10 (1.0**-**1.13)	<.001
Age × log(t)	0.97 (0.96-0.98)	<.001	0.97 (0.96**-**0.98)	<.001
Discharge KPS (per point)			0.99 (0.98**-**1.00)	.018
Extent of resection				
Debulking vs biopsy			0.59 (0.43**-**0.81)	.001
Near-total vs biopsy			0.45 (0.32**-**0.65)	<.001
Gross-total vs biopsy			0.40 (0.30**-**0.53)	<.001
Any complication (yes vs no)			1.23 (0.97**-**1.56)	.093

Bold rows indicate the adjusted estimate for the main exposure of interest: DEX high-dose (≥34 mg) versus low-dose (<34 mg).

## Discussion

We found that cumulative perioperative DEX exposure exceeding 34 mg was associated with reduced overall ­survival in patients with GBM. In our primary total-effect model, which captures the overall clinical impact of DEX across all potential pathways, this association remained significant after adjustment for established baseline prognostic factors. Importantly, the association persisted in a complementary direct-effect model that additionally accounted for postoperative functional status, EOR, and perioperative complications. These findings suggest that the observed survival disadvantage is not fully explained by differences in baseline characteristics or postoperative clinical course and may reflect an effect related to DEX exposure itself.

Age demonstrated a time-dependent association with mortality, with older age conferring a higher hazard early after surgery but a relative attenuation of this effect over longer follow-up.

This pattern is clinically plausible in GBM where early mortality is concentrated among older patients in the immediate postoperative and treatment-initiation phase, whereas long-term outcomes may be increasingly driven by tumor biology and treatment-related factors rather than age alone. Admission KPS was a significant predictor in the total-effect model but lost statistical significance in the direct-effect model. This pattern is consistent with the prespecified DAG structure, in which admission KPS lies upstream of discharge KPS. Because discharge KPS represents a downstream variable that captures postoperative functional recovery, conditioning on it absorbs a substantial portion of the prognostic information carried by admission KPS, thereby attenuating its independent association with survival.

In clinical practice, patients with newly diagnosed GBM frequently receive high initial doses of DEX, often 3 × 4 mg per day or more, maintained for several days until surgery and only gradually tapered thereafter. Moreover, during the course of radiotherapy, DEX is often used as well to manage treatment-related symptoms or restarted in patients in whom it had already been tapered. Given the long biological half-life of DEX with sustained effects on target tissues for approximately 36-72 h, such frequent daily dosing beyond an initial loading phase may not always be necessary.^29^ A treatment strategy consisting of an initial loading dose followed by lower maintenance dosing (e.g., 2-4 mg per day) may be sufficient for symptom control in many cases.

In our cohort, we identified a cumulative perioperative cut-off of 34 mg as significantly associated with worse survival, indicating that even comparatively low total DEX exposure may have a clinically relevant impact on outcome and should prompt careful consideration when prescribing these drugs. Notably, the mean cumulative DEX dose in our cohort was 75.6 ± 44.8 mg, which is more than twice the identified cut-off. Consequently, only 79 patients remained below this threshold, whereas 341 exceeded it, underscoring that in real-world settings, the majority of GBM patients receive higher perioperative DEX doses.

This study is the first to systematically quantify cumulative perioperative DEX exposure during the initial neurosurgical phase, spanning admission to postoperative discharge. Direct comparison of our findings with previous work is challenging because most published studies have assessed DEX use during later stages of the treatment pathway, such as the postoperative period or during adjuvant chemoradiation.^7^^,^^8^^,^^10^ Other investigations examined DEX primarily as a categorical variable rather than as a cumulative dose.^30^ In studies reporting corticosteroid exposure quantitatively, mean daily doses or time-weighted averages were used instead of total perioperative exposure, which limits comparability.^11^^,^^22^^,^^31^ Capturing the full perioperative DEX dose during the neurosurgical stay provides a clearer and more clinically relevant estimate of early steroid exposure.

Survival in patients with GBM is shaped by a multitude of clinical and treatment-related factors, making causal inference inherently challenging, which is an issue well recognized in the literature and addressed in several previous studies.^7–9^^,^^11^ As expected, patients in the high-dose DEX group presented with poorer functional status, reflected by lower admission KPS, and had significantly larger tumor volumes. They also experienced longer hospital stays and a higher rate of overall complications. These differences indicate that higher DEX doses were clinically warranted in patients with greater tumor burden and poorer baseline condition, thereby introducing potential confounding. In contrast, EOR, tumor focality, MGMT promoter methylation status, discharge KPS, and type of adjuvant therapy did not differ significantly between groups.

To account for baseline imbalances and to estimate the independent effect of high-dose DEX exposure, we applied a structured, causally informed modeling framework rather than relying on conventional covariate inclusion, which has varied substantially across prior studies. Within this framework, adjustment sets were defined based on prespecified clinical assumptions. The association between high-dose DEX exposure and reduced survival remained consistent after accounting for baseline prognostic factors as well as postoperative clinical variables. These findings are consistent with prior reports demonstrating an adverse association between DEX administration and survival in GBM.^7^^,^^8^^,^^10^^,^^11^

The correlation between high perioperative DEX exposure and reduced survival in GBM may be explained by numerous potential mechanisms. Preclinical studies suggest that DEX may enhance DNA repair and chemoresistance, promote tumor invasion, proliferation, and angiogenesis, and impair T-cell function while altering the tumor microenvironment, each of which could contribute to worse outcomes in GBM.^8^^,^^13^^,^^15^

Several clinical explanations have also been proposed for the association between DEX use and shorter survival. DEX has been reported to reduce the effectiveness of radiation therapy, immunotherapy, and chemotherapy.^16–18^^,^^32^ In addition, complications related to DEX, such as hyperglycemia, increased infection risk, and other steroid-associated adverse effects, have been linked to poorer outcomes and may contribute indirectly to reduced survival.^19–22^^,^^33^ The controversy surrounding DEX use in GBM remains highly relevant, and multiple aspects of this issue are still unresolved.

### Strengths and Limitations

A strength of this study is the use of a large and well-characterized institutional cohort. This is the first study to provide data on perioperative DEX administration specifically during the initial neurosurgical phase, from admission for surgical evaluation through postoperative discharge. This period is clinically important, as mass effect and neurological deterioration frequently prompt the initiation of higher DEX doses during this interval, yet these data are often poorly documented or not systematically analyzed in previous studies. Our dataset allowed precise quantification of DEX exposure during this interval, and we applied a structured, DAG-informed analytical framework to explicitly define causal assumptions and derive robust adjustment sets, thereby minimizing residual confounding while avoiding overadjustment or inappropriate conditioning on downstream variables.

This study has several limitations. As a retrospective, single-center analysis, it is subject to the typical constraints of observational research, including residual confounding and limited generalizability. Although we adjusted for well-known established clinical and treatment-related confounders, additional unmeasured or incompletely recorded variables may have influenced both DEX administration and survival. Furthermore, while we applied a structured DAG-informed framework to guide covariate selection, the specified DAG reflects our prespecified clinical assumptions regarding the relationships between variables and cannot exclude the possibility of misspecified causal pathways. Our analysis was restricted to the perioperative period, and information on DEX exposure prior to admission or during chemoradiotherapy was not available, limiting the ability to assess the impact of DEX use beyond neurosurgical hospitalization. Missing or incomplete clinical records required the use of a complete-case analysis, which led to the exclusion of some patients and may have introduced selection bias. These limitations should be considered when interpreting the findings of this study.

### Conclusion and Relevance

Higher perioperative DEX exposure was associated with reduced overall survival in patients with GBM, and this association remained consistent after structured adjustment for baseline and postoperative clinical factors. These findings highlight the importance of carefully evaluating the indication for steroid use during neurosurgical care and limiting DEX administration to patients with clear signs of increased intracranial pressure or symptomatic edema. When DEX is required, prompt tapering should be considered. Prospective studies are needed to better account for confounding and to guide evidence-based corticosteroid use in GBM care.

## Supplementary Material

vdag099_Supplementary_Data

## Data Availability

Data will be made available to researchers upon reasonable request.
