# Supplementary material for: Higher perioperative dexamethasone exposure is associated with shorter survival in glioblastoma
Source: Neurooncol Adv. 2026 Apr 13;8(1):vdag099. doi: 10.1093/noajnl/vdag099 (PMC13148246; doi:10.1093/noajnl/vdag099)

Supplemental Material

Supplementary Material 1

DAG specification code (compatible with dagitty R package and dagitty.net)

```
dag {
bb="-6.982,-3.829,6.988,6.268"
"Adjuvant Therapy" [pos="4.400,0.211"]
"Admission GCS" [pos="-4.371,0.226"]
"Admission KPS" [pos="-2.600,3.122"]
"Discharge KPS" [pos="0.291,3.100"]
"Length of stay" [pos="-4.440,3.108"]
"MGMT promotor methylation" [pos="5.946,0.226"]
"Tumor volume" [pos="-0.761,-2.560"]
Age [pos="4.415,3.115"]
Complications [pos="2.001,3.115"]
DEX [exposure,pos="-2.593,0.211"]
EOR [pos="2.750,-2.568"]
OS [outcome,pos="1.131,0.204"]
"Adjuvant Therapy" -> OS
"Admission GCS" -> "Admission KPS"
"Admission GCS" -> DEX
"Admission KPS" -> "Discharge KPS"
"Admission KPS" -> DEX
"Admission KPS" -> EOR
"Admission KPS" -> OS
"Discharge KPS" -> "Adjuvant Therapy"
"Discharge KPS" -> OS
"Length of stay" -> DEX
"MGMT promotor methylation" -> "Adjuvant Therapy"
"Tumor volume" -> "Admission GCS"
"Tumor volume" -> "Admission KPS"
"Tumor volume" -> Complications
"Tumor volume" -> DEX
"Tumor volume" -> EOR
"Tumor volume" -> OS
```

```
Age -> "Adjuvant Therapy"
Age -> Complications
Age -> DEX
Age -> EOR
Age -> OS
Complications -> "Adjuvant Therapy"
Complications -> "Discharge KPS"
Complications -> OS
DEX -> "Discharge KPS"
DEX -> Complications
DEX -> OS
EOR -> "Adjuvant Therapy"
EOR -> "Discharge KPS"
EOR -> OS
}
```

Supplementary Material 2

**Complication**	**Overall** N = 420[1]	**Low-dose (<34 mg)** N = 79[1]	**High-dose (≥34 mg)** N = 341[1]	**p-value**[2]
**Any complication**	109 (27%)	9 (12%)	100 (30%)	**0.003**
**Neurological (any)**	73 (18%)	6 (8.0%)	67 (20%)	**0.022**
Missing	10 (2.4%)	4 (5.1%)	6 (1.8%)	
Seizure	26 (6.2%)	3 (3.8%)	23 (6.7%)	
New or worsened neurological deficit	32 (7.6%)	4 (5.1%)	28 (8.2%)	
Intracranial hemorrhage	14 (3.3%)	0 (0%)	14 (4.1%)	
Hydrocephalus	6 (1.4%)	0 (0%)	6 (1.8%)	
Herniation / transtentorial mass effect	1 (0.2%)	0 (0%)	1 (0.3%)	
Other	24 (5.7%)	3 (3.8%)	21 (6.2%)	
**Systemic (any)**	56 (14%)	6 (8.0%)	50 (15%)	0.16
Missing	12 (2.9%)	4 (5.1%)	8 (2.3%)	
Surgical site infection	6 (1.4%)	1 (1.3%)	5 (1.5%)	
Pneumonia	3 (0.7%)	0 (0%)	3 (0.9%)	
Urinary tract infection	4 (1.0%)	0 (0%)	4 (1.2%)	
Deep vein thrombosis	6 (1.4%)	0 (0%)	6 (1.8%)	
Pulmonary embolism	6 (1.4%)	0 (0%)	6 (1.8%)	
Steroid-induced psychosis	4 (1.0%)	2 (2.5%)	2 (0.6%)	
Adrenal insufficiency	2 (0.5%)	1 (1.3%)	1 (0.3%)	
Other	43 (10%)	4 (5.1%)	39 (11%)	

[1] n (%)

[2] Pearson's Chi-squared test

Supplementary Material 3

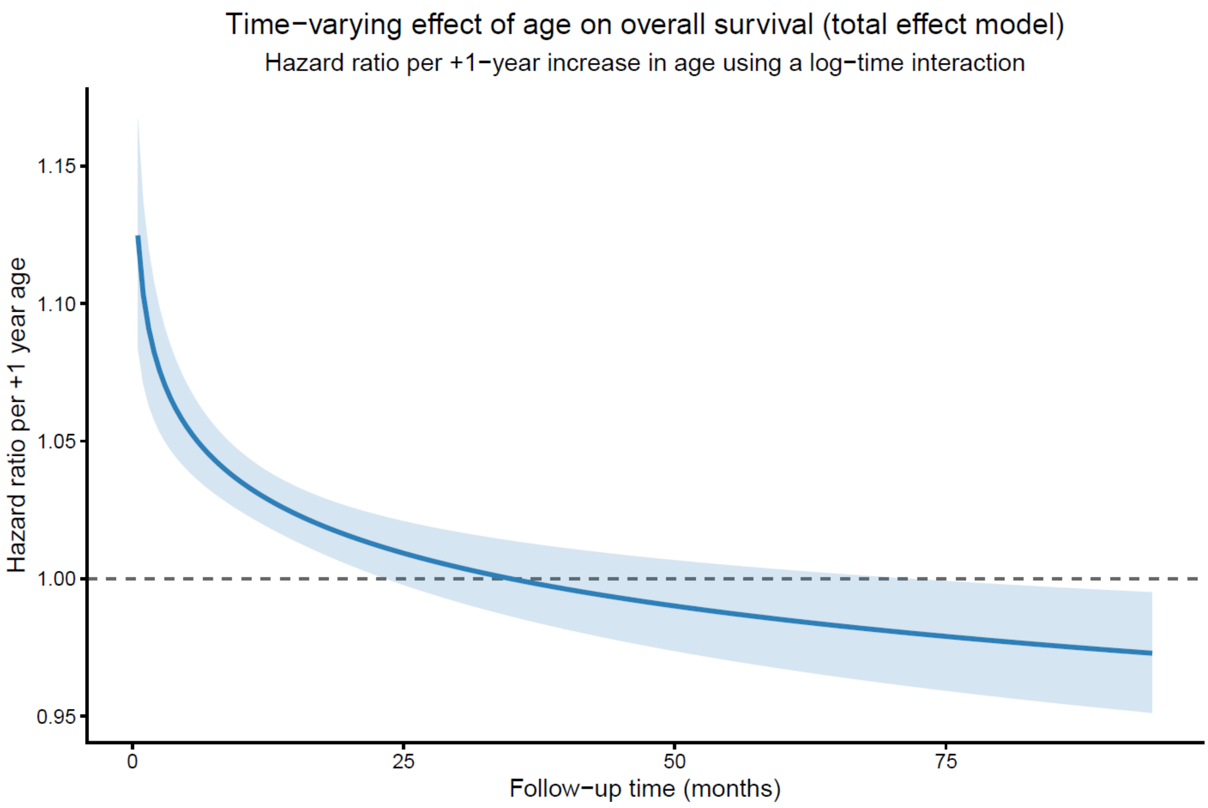

Supplement: vdag099_Supplementary_Data [file vdag099_supplementary_data.zip › 29-Apr-2026_025402_Supplemental_Material.pdf]
