# Supplementary material for: Higher perioperative dexamethasone exposure is associated with shorter survival in glioblastoma
Source: Neurooncol Adv. 2026 Apr 13;8(1):vdag099. doi: 10.1093/noajnl/vdag099 (PMC13148246; doi:10.1093/noajnl/vdag099)

**Supplemental Material**

**Supplementary Material 1**

DAG specification code (compatible with dagitty R package and dagitty.net)

dag {

bb="-6.982,-3.829,6.988,6.268"

"Adjuvant Therapy" [pos="4.400,0.211"]

"Admission GCS" [pos="-4.371,0.226"]

"Admission KPS" [pos="-2.600,3.122"]

"Discharge KPS" [pos="0.291,3.100"]

"Length of stay" [pos="-4.440,3.108"]

"MGMT promotor methylation" [pos="5.946,0.226"]

"Tumor volume" [pos="-0.761,-2.560"]

Age [pos="4.415,3.115"]

Complications [pos="2.001,3.115"]

DEX [exposure,pos="-2.593,0.211"]

EOR [pos="2.750,-2.568"]

OS [outcome,pos="1.131,0.204"]

"Adjuvant Therapy" -> OS

"Admission GCS" -> "Admission KPS"

"Admission GCS" -> DEX

"Admission KPS" -> "Discharge KPS"

"Admission KPS" -> DEX

"Admission KPS" -> EOR

"Admission KPS" -> OS

"Discharge KPS" -> "Adjuvant Therapy"

"Discharge KPS" -> OS

"Length of stay" -> DEX

"MGMT promotor methylation" -> "Adjuvant Therapy"

"Tumor volume" -> "Admission GCS"

"Tumor volume" -> "Admission KPS"

"Tumor volume" -> Complications

"Tumor volume" -> DEX

"Tumor volume" -> EOR

"Tumor volume" -> OS

Age -> "Adjuvant Therapy"

Age -> Complications

Age -> DEX

Age -> EOR

Age -> OS

Complications -> "Adjuvant Therapy"

Complications -> "Discharge KPS"

Complications -> OS

DEX -> "Discharge KPS"

DEX -> Complications

DEX -> OS

EOR -> "Adjuvant Therapy"

EOR -> "Discharge KPS"

EOR -> OS

}

**Supplementary Material 2**


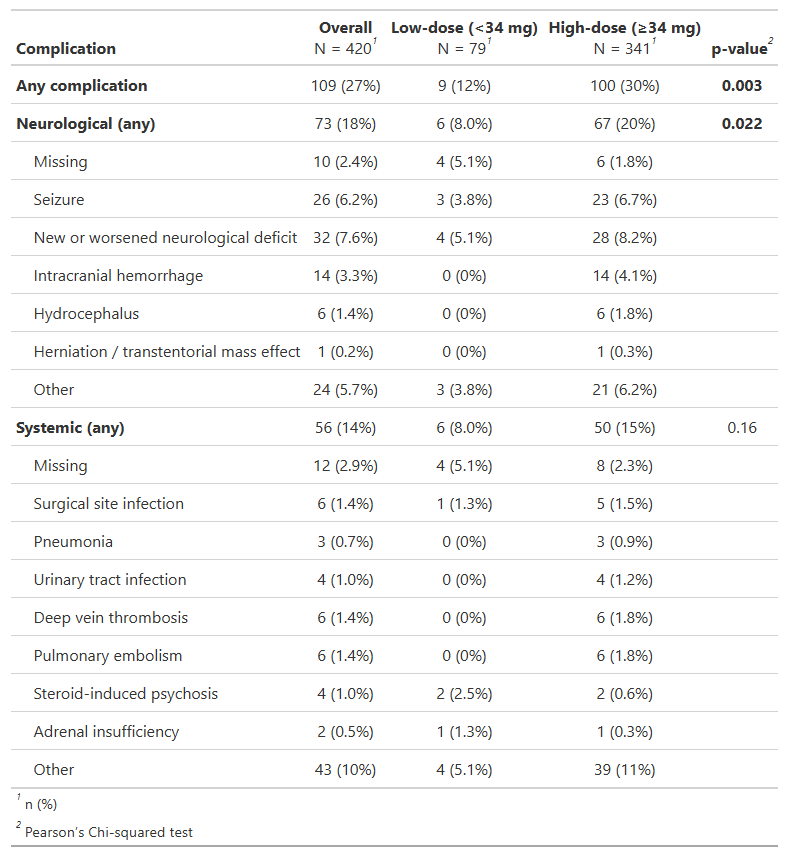


**Supplementary Material 3**


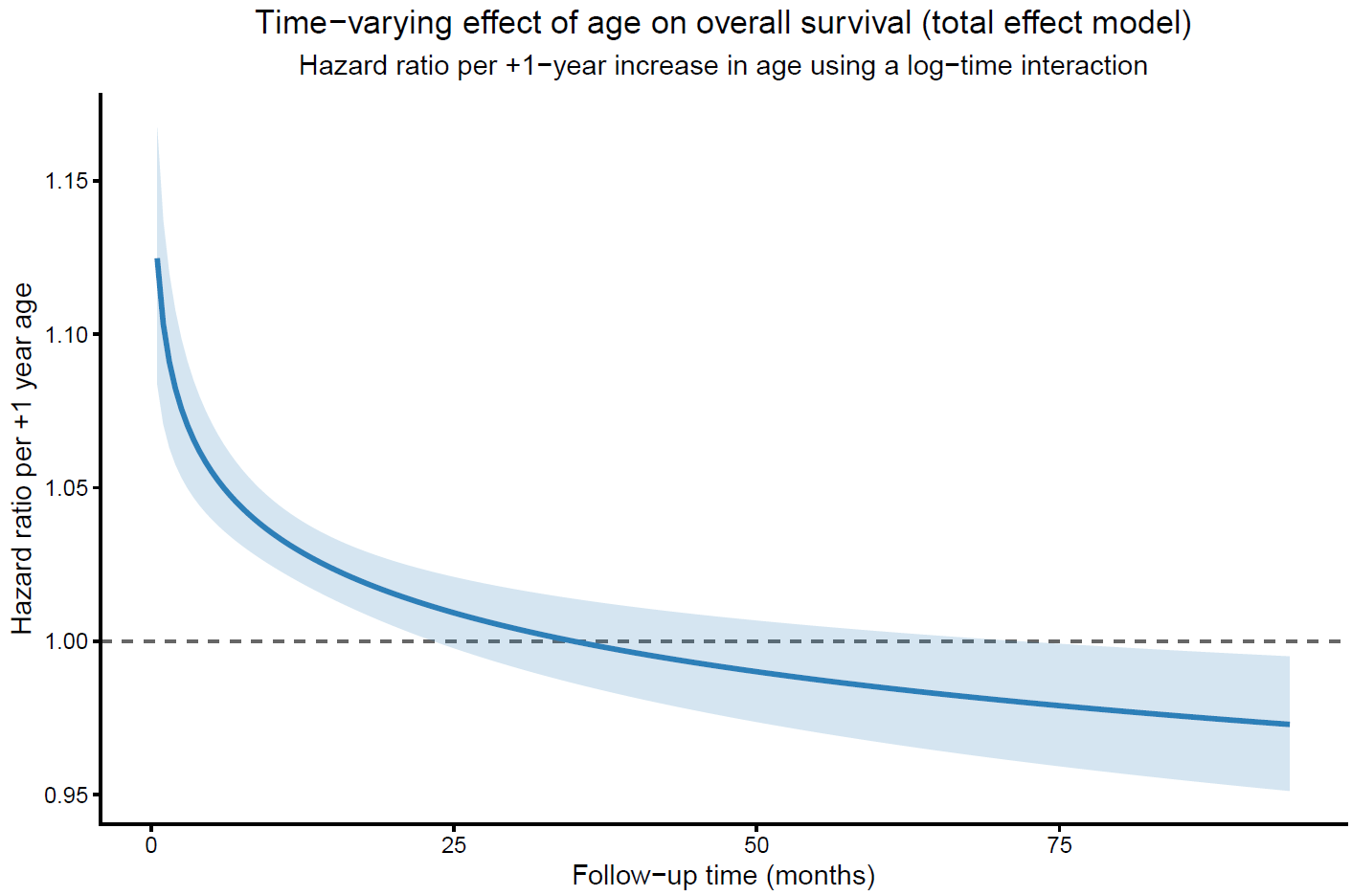

Supplement: vdag099_Supplementary_Data [file vdag099_supplementary_data.zip › Supplemental_Material.docx]
